# Emergency Physicians’ Perceptions, Knowledge, and Attitudes Toward Family Presence During Resuscitation in the Emergency Department: A Multicenter Survey-Based Cross-Sectional Study

**DOI:** 10.7759/cureus.80612

**Published:** 2025-03-15

**Authors:** Asseil A Bossei, Hanan A Al Zahrani, Faisal A Bossei, Sufana M Saadi, Ahmed S Alsaedi, Abdulrahman Q Al Sulami, Emad H Al Asmari, Ahmad A Aalam, Imad M Khojah

**Affiliations:** 1 Emergency Medicine, King Abdullah Medical Complex, Jeddah, SAU; 2 Emergency Medicine, King Abdulaziz Medical City, Jeddah, SAU; 3 Emergency Medicine, Al-Noor Specialist Hospital, Makkah Health Cluster, Makkah, SAU; 4 Emergency Medicine, Al Salama Hospital, Jeddah, SAU; 5 Internal Medicine, King Abdullah Medical Complex, Jeddah, SAU; 6 Emergency Medicine, King Abdulaziz Hospital, Jeddah, SAU; 7 General Practice, Al-Hijra Primary Healthcare Center, Jeddah, SAU; 8 Emergency Medicine, Faculty of Medicine, King Abdulaziz University Hospital, Jeddah, SAU; 9 Emergency Medicine, Faculty of Medicine, King Abdulaziz University, Jeddah, SAU

**Keywords:** cardiopulmonary resuscitation, emergency department, emergency medicine, family presence during resuscitation, fpdr policies, saudi arabia

## Abstract

Background: Cardiac arrest remains a significant public health issue, with high incidence rates in both in-hospital and out-of-hospital settings. The practice of family presence during resuscitation (FPDR) has gained attention for its potential benefits to patients and their families. This study evaluates the perceptions, knowledge, and attitudes of emergency physicians (EPs) regarding FPDR in the emergency department (ED) and aims to inform policy development at King Abdulaziz University Hospital in Jeddah, Saudi Arabia.

Methods: A multicenter cross-sectional study was conducted from January to April 2023, surveying EPs from multiple EDs across the western region of Saudi Arabia. Participants, certified in basic (BLS) or advanced life support (ALS), completed an anonymous online survey adapted from previous studies.

Results: Our study surveyed 122 EPs, with 112 completing the survey. Of the participants, 49.1% were aged 25-29 years, 61.6% were men, and 58.9% had 1-4 years of work experience. Awareness of FPDR was reported by 67.9% (n = 76) of participants. Only 3.6% (n = 4) had a policy allowing FPDR, while 6.3% (n = 7) had a policy prohibiting it. Additionally, 49.1% (n = 55) supported implementing an FPDR policy. Awareness of FPDR was significantly higher among younger, male, and more experienced physicians (p < 0.05). Higher perception and practice scores were observed among those aware of FPDR, those who had participated in CPR with a family member, and those without a prohibiting policy (p < 0.05).

Conclusion: EPs in the western region of Saudi Arabia generally support FPDR, recognizing its potential benefits. However, concerns about its impact on performance and medicolegal issues warrant further exploration. To implement effective FPDR policies, these concerns must be addressed, along with efforts to promote awareness and training. Future research should expand to include broader healthcare settings and multidisciplinary teams to develop comprehensive, evidence-based guidelines.

## Introduction

Cardiac arrest is a critical medical emergency with profound implications not only for patient survival but also for the psychological well-being of their relatives [1]. Family members who witness resuscitation efforts often experience intense emotional distress, which can manifest as anxiety, post-traumatic stress, or a sense of exclusion from the decision-making process. However, studies suggest that allowing family presence during cardiopulmonary resuscitation (CPR) may provide emotional closure, improve understanding of the patient’s condition, and foster trust in medical teams.

Cardiovascular diseases continue to be a major cause of mortality worldwide, prompting the adoption of machine learning approaches and other innovative techniques to improve both clinical and psychological outcomes [2]. Cardiac arrest, in particular, represents one of the most pressing public health concerns, with high incidence rates in both in- and out-of-hospital settings. While a significant proportion of cardiac arrests occur outside of medical facilities, the burden of in-hospital cardiac arrest (IHCA) is substantial. Recent studies report that approximately 300,000 IHCAs occur annually in the United States (US), underscoring the critical need for effective resuscitation strategies and family-inclusive policies in emergency settings [1,3]. Additionally, emerging evidence highlights the role of advanced hemodynamic monitoring techniques, such as perfusion index and end-tidal carbon dioxide levels, in improving resuscitation outcomes and guiding CPR interventions [3]. Despite ongoing debates, the practice of permitting family presence during resuscitation (FPDR) remains controversial among emergency medicine (EM) physicians. Factors such as provider discomfort, concerns over medicolegal risks, and potential disruptions to the resuscitation process contribute to varying attitudes toward this practice.

CPR is a crucial emergency procedure that significantly enhances the prognosis of many patients. Statistics from the National Registry of Cardiopulmonary Resuscitation reveal that only 9%-11% of CPRs performed in hospital settings take place within the emergency department (ED) [4]. In-hospital CPR is typically carried out by either a multidisciplinary advanced life support (ALS) team or a basic life support (BLS) team. These teams are responsible for maintaining clear airways, providing adequate rescue breaths, administering chest compressions, and delivering early defibrillation to patients in cardiac arrest [4,5].

The American Heart Association, along with other global organizations, highlights and advocates for the concept of FPDR, defined as "the presence of one or more family members or significant others in a location that enables visible or physical contact with the patient during invasive procedures or CPR" [6]. This practice has garnered significant attention due to its potential benefits for both the patient and their family, including emotional closure, improved trust in medical teams, and reduced psychological distress among relatives. Additionally, FPDR aligns with patient-centered care principles, emphasizing the importance of transparency and family involvement in critical medical interventions. Advocates of FPDR argue that allowing family members to be present during such critical moments can provide emotional support to both the patient and their loved ones by fostering a sense of familiarity and comfort in a highly stressful situation [6,7]. Family presence has been associated with reduced psychological distress, as it allows relatives to witness medical efforts firsthand, promoting transparency and trust in healthcare providers. Additionally, previous research suggests that FPDR may lower the incidence of post-traumatic stress disorder (PTSD), anxiety, and depression among family members of resuscitated patients. A review by Clark et al. found that relatives who were allowed to witness CPR had a significantly lower rate of PTSD symptoms three months post-resuscitation compared to those who were not present [7]. 

Additionally, it offers family members the opportunity to witness the medical efforts being made, which can aid in their understanding and acceptance of the patient's condition and the outcomes of the resuscitative efforts [8,9]. Research suggests that FPDR can also support the grieving process by providing a sense of closure and understanding, as family members can witness firsthand the heroic efforts made to save their loved one [9]. Despite these potential benefits, FPDR remains a topic of debate within the medical community, with concerns about its impact on healthcare providers' performance, the potential for increased stress or interference, and the appropriateness of such practices in all clinical situations [8,9]. In Saudi Arabia, cultural norms and limited FPDR policies may shape physician perspectives uniquely.

This study aims to evaluate the perceptions, knowledge, and attitudes of EPs regarding FPDR in the ED.

## Materials and methods

Study design and population

This is a multicenter, cross-sectional study that examines EPs' perceptions and attitudes toward FPDR in ED CPR cases in the western region of Saudi Arabia, conducted from January to April 2023. The study population included EPs working full-time at local EDs across the western region, spanning all residency and career levels, from residents to consultants/attendings. The study included licensed healthcare professionals, specifically emergency medicine physicians and nurses, who held a valid BLS or ALS certification at the time of enrollment and provided informed consent. Exclusion criteria encompassed individuals without a valid BLS or ALS certification, those with expired or unverified certifications, participants with incomplete data, and individuals who declined to participate.

Data collection

An anonymous, self-administered online survey was distributed via Google Forms over a one-month period to emergency medicine (EM) physicians and nurses working in hospitals in the western region of Saudi Arabia. Eligible participants were identified based on their active practice in emergency medicine, verified through hospital staff records and professional medical associations. The invitations were sent through official hospital email lists, professional networks, and direct outreach via medical organizations. The survey questions were adapted from previous studies [7]. Given the high reliability and validity ratings of the questionnaires by Tomlinson et al. [6] and Kianmehr et al. [10], items from these studies were incorporated into this survey. A Cronbach’s alpha reliability coefficient was calculated using IBM SPSS Statistics for Windows, Version 26.0 (Released 2019; IBM Corp., Armonk, NY, US) based on the 18 standardized items in the questionnaire, yielding a value of 0.789, which indicates acceptable reliability.

The questionnaire was divided into three sections. The first section collected demographic data, including age, gender, level of expertise, work experience, hospital type, and the number of CPR cases led per month. The second section consisted of four questions regarding the participants’ knowledge and experience of FPDR. The third section included 18 items assessing participants' perceptions and practices regarding FPDR. These items were measured using a five-point Likert scale, with scores ranging from 1 (strongly disagree) to 5 (strongly agree). Reverse-coded questions were scored from 1 to 5. The total score for EPs' perception and practice of FPDR ranged from 18 to 90, with 18 indicating the lowest knowledge score and 90 the highest knowledge score. The questionnaire is provided as a supplementary file (Appendix 1) to enhance transparency and facilitate future research replication.

Ethical approval

Ethical approval for the study was obtained from the Research Ethics Committee of King Abdulaziz University Hospital, Jeddah, Saudi Arabia (NCBE registration number: HA-02-J-008). To ensure adequate statistical power, the sample size was initially calculated to be 168 ED physicians, based on a medium effect size (Cohen's d = 0.5) observed in previous studies examining healthcare providers' perceptions of FPDR. Additionally, for a finite population of 250, with a 95% confidence interval (p = 0.5) and a margin of error of 5%, the final required sample size was estimated to be 152 participants [11]. However, we successfully collected data from 112 participants. A post hoc power analysis confirmed that our sample size provides sufficient statistical power to detect meaningful effects, indicating that further data collection was unnecessary.

Data analysis

Data analysis was conducted using IBM SPSS Statistics for Windows, Version 26.0 (Released 2019; IBM Corp., Armonk, NY, US). To examine correlations between variables, qualitative data were quantified as numerical values and percentages and analyzed using the chi-squared test (χ^2^). Quantitative data were expressed as means and standard deviations (mean ± SD). Non-parametric variables were assessed using the Mann-Whitney and Kruskal-Wallis tests. Spearman's rank correlation test was used for analyzing relationships between variables, with statistical significance set at p < 0.05.

## Results

Demographic Data

Our study surveyed 122 EPs, of whom 112 completed the survey. Ten were excluded as they did not meet the inclusion criteria. Table 1 presents the participants' demographic characteristics, showing that 55 (49.1%) of the surveyed EM physicians were aged 25-29 years; of these, 69 (61.6%) were men, 23 (20.5%) were in residency PGY 4, and 66 (58.9%) had 1-4 years of work experience. Forty-three (38.4%) worked at academic hospitals, and 61 (54.5%) led 1-5 CPR cases per month.

**Table 1:** Distribution of studied EM physicians according to their demographics and work data. PGY: postgraduate year.

Variable	Are you aware of the family presence during resuscitation (FPDR) concept?		p-value
	No, n (%)	Yes, n (%)	
Age (years)			0.004
25-29	27 (75)	28 (36.8)	
30-34	6 (16.7)	27 (35.5)	
35-39	2 (5.6)	6 (7.9)	
40-44	0 (0.0)	10 (13.2)	
45-49	0 (0.0)	4 (5.3)	
50-55	1 (2.8)	1 (1.3)	
Gender			0.941
Female	14 (38.9)	29 (38.2)	
Male	22 (61.1)	47 (61.8)	
Level of expertise			<0.001
Residency PGY 1	0 (0.0)	5 (6.6)	
Residency PGY 2	10 (27.8)	5 (6.6)	
Residency PGY 3	5 (13.9)	8 (10.5)	
Residency PGY 4	14 (38.9)	9 (11.8)	
Senior registrar	2 (5.6)	14 (18.4)	
Specialist/registrar	2 (5.6)	19 (25)	
Consultant	3 (8.3)	16 (21.1)	
Work experience (years)			0.01
1-4	30 (83.3)	36 (47.4)	
5-8	3 (8.3)	24 (31.6)	
8-11	1 (2.8)	6 (7.9)	
12-15	1 (2.8)	4 (5.3)	
>15	1 (2.8)	6 (7.9)	
Number of cardiopulmonary resuscitation (CPR) cases you lead per month			0.008
1-5	18 (50)	43 (56.6)	
6-10	6 (16.7)	19 (25)	
11-15	1 (2.8)	6 (7.9)	
16-20	0 (0.0)	2 (2.6)	
>20	0 (0.0)	2 (2.6)	
None per month	11 (30.6)	4 (5.3)	

Outcomes

**Figure 1:**
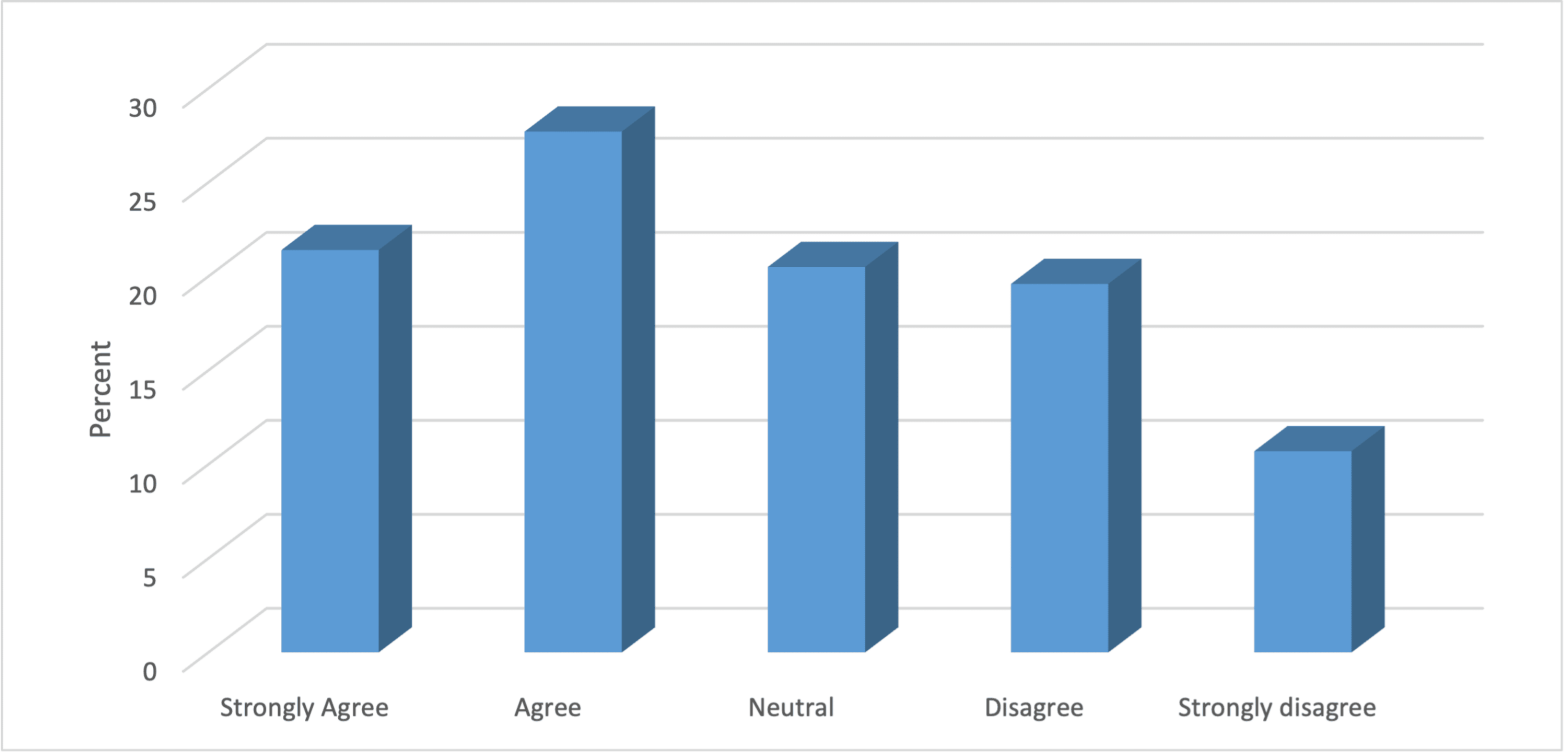
Percentage distribution of EM physicians according to their support in implementing a policy that allows FPDR in their institution.

Most EM physicians (n = 76, 67.9%) were aware of the FPDR concept. Additionally, 73 (65.2%) had participated in CPR with a family member present. Of the 24 hospitals, only four (3.6%) had a written policy in their department/hospital allowing FPDR, while seven (6.3%) had a policy prohibiting it. Regarding policy support, 55 (49.1%) agreed or strongly agreed with implementing a policy allowing FPDR in their institution. Awareness of the FPDR concept was significantly higher among men, specialists/registrars, those with 1-4 years of work experience, and those leading 1-5 CPR cases per month (p < 0.05) (Table 2 and Figure 1).

**Table 2:** Distribution of EM physicians according to their knowledge and experience of FPDR.

	I don't know	No	Yes
Are you aware of the family presence during resuscitation (FPDR) concept?	0	36 (32.1)	76 (67.9)
Have you participated in CPR during which a family member was present?	0	39 (34.8)	73 (65.2)
Do you have a written policy in your department/hospital allowing FPDR?	76 (67.9)	32 (28.6)	4 (3.6)
Do you have a written policy in your department/hospital prohibiting FPDR?	73 (65.2)	32 (28.6)	7 (6.3)

The mean score of physicians' perception and practice was significantly higher among male physicians (p < 0.05) (Table 3). No significant relationship was found between the mean total score and other demographics or work data (p > 0.05). However, Table 4 shows that the mean score of physicians’ perception and practice was significantly higher among physicians aware of the FPDR concept (p < 0.05). Similarly, the mean total score was significantly higher among those who had participated in CPR with a family member present (p < 0.05). A significantly higher mean total score was also observed among those without a written policy prohibiting FPDR in their department/hospital (p < 0.05).

**Table 3:** Relationship between EM physicians' awareness of the family presence during resuscitation (FPDR) concept and their demographics and work data. PGY: postgraduate year.

	Are you aware of the family presence during resuscitation (FPDR) concept?		χ^2^	df	Effect size	p-value
Variable	No	Yes			Value (significance)	
	n, (%)	n, (%)				
Age (years)						
25-29	27 (75)	28 (36.8)	17.3	5	0.007 (0.94)	0.004
30-34	6 (16.7)	27 (35.5)				
35-39	2 (5.6)	6 (7.9)				
40-44	0 (0.0)	10 (13.2)				
45-49	0 (0.0)	4 (5.3)				
50-55	1 (2.8)	1 (1.3)				
Gender						
Female	14 (38.9)	29 (38.2)	0.006	1	0.39 (0.004)	0.941
Male	22 (61.1)	47 (61.8)				
Level of expertise						
Residency PGY 1	0 (0.0)	5 (6.6)	29.59	6	0.51 (<0.001)	<0.001
Residency PGY 2	10 (27.8)	5 (6.6)				
Residency PGY 3	5 (13.9)	8 (10.5)				
Residency PGY 4	14 (38.9)	9 (11.8)				
Senior registrar	2 (5.6)	14 (18.4)				
Specialist/registrar	2 (5.6)	19 (25)				
Consultant	3 (8.3)	16 (21.1)				
Work experience (years)						
1-4	30 (83.3)	36 (47.4)	13.22	4	0.34 (0.01)	0.01
5-8	3 (8.3)	24 (31.6)				
8-11	1 (2.8)	6 (7.9)				
12-15	1 (2.8)	4 (5.3)				
>15	1 (2.8)	6 (7.9)				
Number of cardiopulmonary resuscitation (CPR) cases you lead per month						
1-5	18 (50)	43 (56.6)	15.54	5	0.37 (0.008)	0.008
6-10	6 (16.7)	19 (25)				
11-15	1 (2.8)	6 (7.9)				
16-20	0 (0.0)	2 (2.6)				
>20	0 (0.0)	2 (2.6)				
None per month	11 (30.6)	4 (5.3)				

**Table 4:** Relationship between mean total physicians' perception and practice score and their knowledge and experience of FPDR.

Variable	Total physicians' perception and practice score (mean ± SD)	p-value
Are you aware of the family presence during resuscitation (FPDR) concept?		0.002
No	47.3 ± 8.88	
Yes	53.86 ± 10.02	
Have you participated in CPR during which a family member was present?		0.006
No	48.15 ± 8.62	
Yes	53.68 ± 10.37	
Do you have a written policy in your department/hospital allowing FPDR?		0.912
I don't know	51.44 ± 9.65	
No	52.71 ± 11.3	
Yes	50 ± 10.8	
Do you have a written policy in your department/hospital prohibiting FPDR?		0.006
I don't know	51.56 ± 10.5	
No	54.46 ± 8.13	
Yes	41.42 ± 7.8	

**Figure 2:**
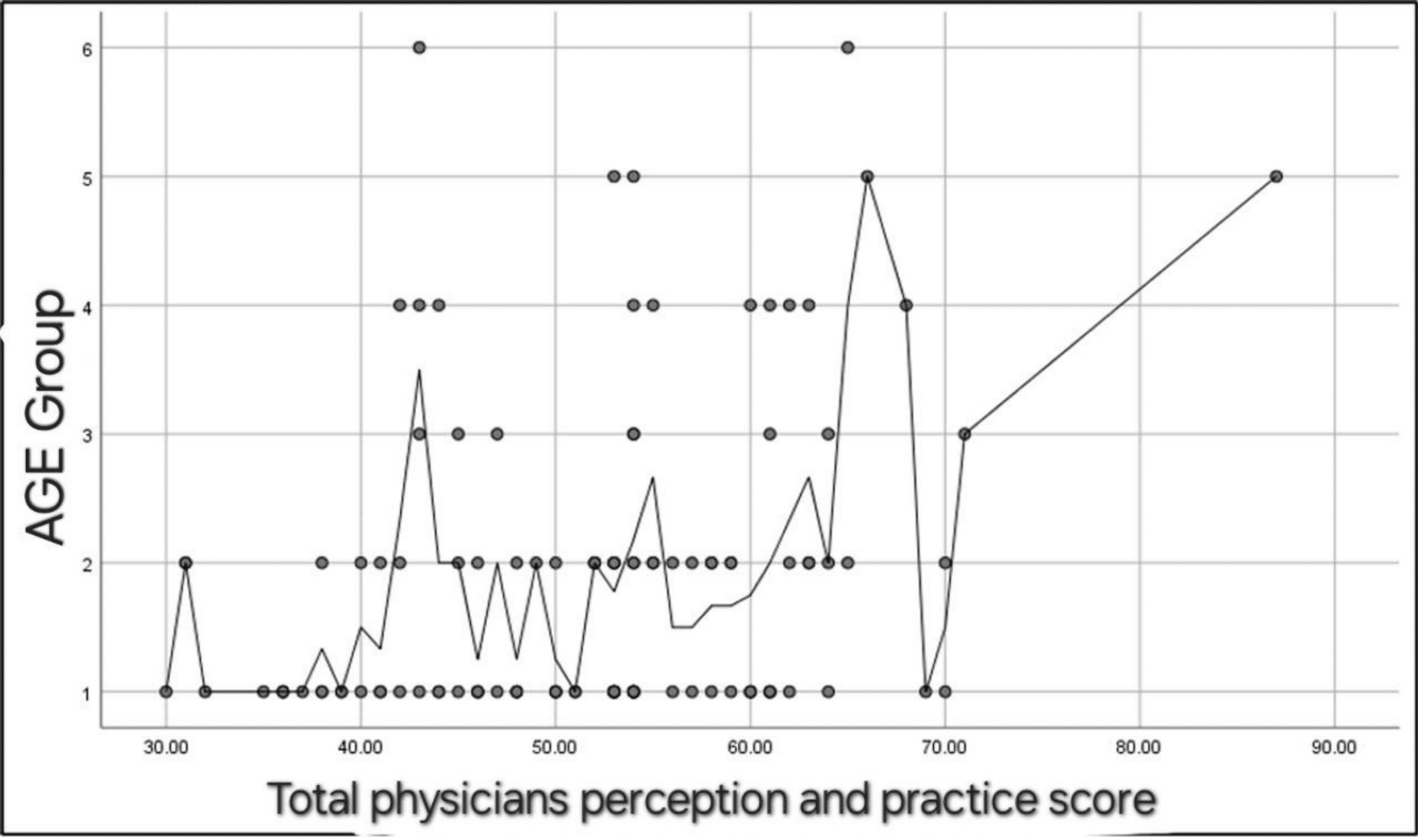
Spearman's correlation between the total physicians' perception and practice score and their age. N.B.: r =0.29, p = 0.002). Age groups: (1) 25-29 years old, (2) 30-34 years old, (3) 35-39 years old, (4) 40-44 years old, (5) 45-49 years old, (6) 50-55 years old.

**Figure 3:**
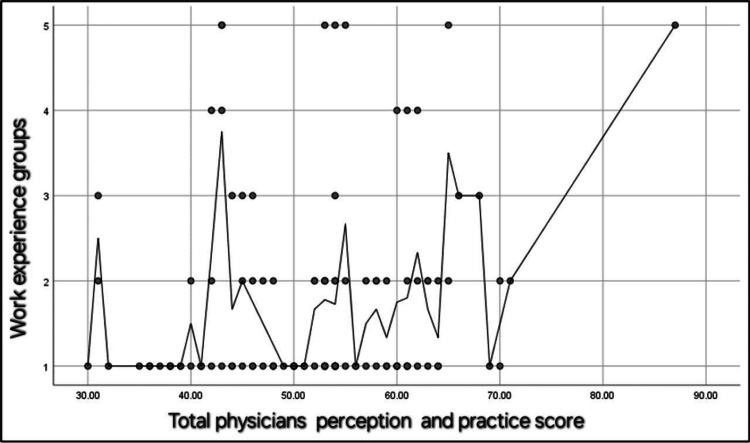
Spearman's correlation between the total physicians' perception and practice score and their work experiences in years. N.B.: r =0.18, p = 0.04. Work experience groups: (1) 1-4 years of experience, (2) 5-8 years of experience, (3) 8-11 years of experience, (4) 12-15 years of experience, (5) more than 15 years of experience.

Additionally, there was a significant positive correlation between the total physicians' perception and practice score and their age (r = 0.29, p = 0.002) (Figure 2). A significant positive correlation was also found between the total physicians' perception and practice score and their work experience in years (r = 0.18, p = 0.04) (Figure 3).

## Discussion

Qualitative and survey-based studies have shown that FPDR positively impacts families' psychological well-being [12-14], prompting numerous professional organizations to recommend policies supporting FPDR [15-17]. Despite these endorsements, many healthcare professionals continue to oppose FPDR, citing potential disruptions to resuscitation efforts and risks to patient safety [18].

Our cross-sectional study revealed that EM physicians' perceptions, knowledge, and attitudes toward FPDR in the ED were predominantly positive and statistically significant. This finding contrasts with the study by Al Bshabshe et al. [19], where physicians largely opposed FPDR. This discrepancy may be attributed to various cultural, institutional, and educational differences between the study populations. Furthermore, our data indicated a positive correlation between age and experience on physicians' perceptions of FPDR, as illustrated in Figure 2 and Figure 3. This suggests that more experienced and older physicians might have developed a greater appreciation for the benefits of FPDR over time. A literature review of related studies was conducted and is summarized in Table 5. Demir [20] found that 82.6% of the participants did not support FPDR. However, in our study, 67.9% of the participants were aware of and supported FPDR.

**Table 5:** A literature review of the related studies.

Study	Number of participants	Findings
Demir [20]	144	No benefits were noted for family presence during resuscitation. The majority (82.6%) did not support it, with the most common concerns being that family members would interfere with the team’s activities (56.3%) and that resuscitation could be too traumatic for families (43.6%).
Gold et al. [21]	521	Half of the participants believed that family presence during resuscitation was beneficial for parents, with two-thirds indicating that parents would want the option. However, a key barrier identified was the potential for family presence to intimidate the resident physician.
Booth et al. [22]	162	Participants highlighted several benefits of family presence during resuscitation, including helping family members accept that everything possible was done (48%), facilitating acceptance of death (48%), and aiding in the grieving process (38%). However, barriers such as concerns about family members becoming distressed and interfering with the resuscitation, fear of litigation, lack of space, and insufficient chaperones were also identified.

A significant majority of EM physicians (67.9%) demonstrated awareness of FPDR. The findings revealed that up to 72% of respondents supported implementing a family facilitator role to enhance the practice of FPDR. Soleimanpour et al. reported that 35.7% of respondents favored allowing family presence during CPR, while 42.2% were against it [23]. Despite this awareness, there exists an antagonistic policy toward FPDR in many hospitals. Specifically, only 6.3% of the respondents reported a written policy in their department or hospital explicitly prohibiting FPDR. Conversely, there appears to be a significant lack of knowledge regarding written policies supporting FPDR, with only 3.6% of respondents indicating such a policy existed. This lack of formal policies may contribute to uncertainty and inconsistency in the application of FPDR practices across institutions.

Age has often been considered a factor influencing attitudes toward family presence during CPR. While a previous cross-sectional study found no correlation between age and acceptance of this practice [23], our study revealed a significant relationship, suggesting a potential change in attitudes or differing population dynamics.

Approximately 65.2% have been in a resuscitation attempt with a family member present. However, other studies conducted on nurses revealed that more than half of the nurses have never been in a resuscitation attempt with a family member present [24,25].

Notably, 49.1% of participants agreed or strongly agreed on the importance of implementing a policy allowing FPDR in their institution. Implementing such policies could standardize the practice, provide clear guidelines, and potentially improve patient and family outcomes during resuscitation efforts [26-28].

Additionally, our study underscored the importance of having dedicated and trained personnel to accompany family members during CPR. This recommendation aligns with the European Resuscitation Council Guidelines of 2021 [29], which advocate for the presence of a designated healthcare professional to provide explanation and comfort to family members present during resuscitation. This approach ensures that family members receive the necessary support and information during critical moments, which can help mitigate the emotional impact of witnessing a resuscitation.

Furthermore, the presence of trained personnel can facilitate better communication and reduce the potential for misunderstandings or conflicts during resuscitation efforts. It also allows the medical team to focus on the resuscitation process, knowing that the family members are being supported and informed by a dedicated professional. This holistic approach to resuscitation not only benefits the family members but also enhances the overall quality of care provided in the ED. The inconclusive findings of the meta-analysis of da Silva Barreto [30] highlight the complexity of FPDR, necessitating careful consideration when integrating it into routine practice. 

Strengths and limitations

Although the study contributes to a better understanding of the barriers to widespread acceptance of FPDR, larger multicenter studies need to be conducted to clearly demonstrate the extent and significance of regional and cultural variations in physicians' perceptions, knowledge, and attitudes toward this critical practice issue in emergency medicine departments. There is also a need to study the perspectives of other healthcare providers toward FPDR. Additionally, self-reported measures (questionnaires) in public surveys that collect data are more susceptible to response and recall biases.

## Conclusions

The majority of EPs in the western region of Saudi Arabia are acquainted with the concept of FPDR and generally hold a favorable attitude toward this practice. However, some physicians have expressed reservations about FPDR, citing concerns regarding potential negative impacts on performance or medicolegal issues. These areas warrant further investigation for a more comprehensive understanding. Finally, effective implementation of FPDR policies necessitates consideration of numerous variables to enhance outcomes for patients, their families, and healthcare providers alike.

## Appendices

Appendix 1

First Section

Gender:

Male

Female

Age:

25-29 years old

30-34 years old

35-39 years old

40-44 years old

45-49 years old

50-55 years old

>55 years old

Level of expertise:

Residency PGY 1

Residency PGY 2

Residency PGY 3

Residency PGY 4

Specialist/registrar

Senior registrar

Consultant

Work experience:

1-4 years

5-8 years

8-11 years

12-15 years

>15 years

Hospital:

King Abdulaziz University Hospital

King Abdulaziz Medical City, National Guard Jeddah

King Abdulla Medical Complex, Jeddah

East Jeddah Hospital

International Medical Center

King Fahad Armed Forces Hospital

King Faisal Specialist Hospital and Research Centre

King Abdullah Medical City, Makkah

Security Forces Hospital, Makkah

King Abdulaziz Specialist Hospital, Taif

Alhada Armed Forces Hospital, Taif

Other:

How many CPR cases do you lead per month:

1-5

6-10

11-15

16-20

>20

None per month

BLS license status:

Active

Expired

Not certified

ALS license status:

Active

Expired

Not certified

*Second Section* 

Are you aware of the FPDR concept?    

Yes

No

Have you participated in CPR during which a family member was present?

Yes

No

Do you have a written policy in your department/hospital allowing FPDR?    

Yes

No

I don't know

Do you have a written policy in your department/hospital prohibiting FPDR?    

Yes

No

I don't know

*Third Section* 

Choose one (strongly agree, agree, neutral, don’t agree, strongly don’t agree)

1. Do you support implementing a policy allowing FPDR in your institution?

2. FPDR is a patient/family right

3. Family members should have the option to attend CPR for adult patients

4. Family members should have the option to attend CPR for pediatric patients

5. FPDR interferes with patient CPR (family may request to continue or to terminate CPR)

6. FPDR decreases family anger toward members of the code team

7. Family members may witness errors or misinterpret some actions during resuscitation

8. FPDR can cause psychological stress/traumatic experiences for the family members

9. FPDR can aid in the grieving process for family members

10. FPDR keeps family members updated about the progress of resuscitation

11. FPDR needs adequate space in the resuscitation room

12. FPDR needs dedicated and trained personnel to accompany family members

13. FPDR is stressful for members of the code team

14. FPDR may pose a physical threat to members of the code team

15. FPDR increases fear of complaints/litigations against members of the code team

16. FPDR may breach patient confidentiality

17. FPDR will motivate members of the code team to manage the patient in a more humane manner (avoid black humor)

18. FPDR impedes training of junior staff during CPR
